# Impact of age on CD4 recovery and viral suppression over time among adults living with HIV who initiated antiretroviral therapy in the African Cohort Study

**DOI:** 10.1186/s12981-020-00323-x

**Published:** 2020-11-12

**Authors:** Emmanuel Bahemana, Allahna Esber, Nicole Dear, Kavitha Ganesan, Ajay Parikh, Domonique Reed, Lucas Maganga, Samoel Khamadi, Mucho Mizinduko, Anange Lwilla, Dorothy Mkondoo, Gwamaka Mwaisanga, Nancy Somi, John Owouth, Jonah Maswai, Francis Kiweewa, Michael Iroezindu, Julie A. Ake, Trevor A. Crowell, Victor G. Valcour, Christina S. Polyak, the AFRICOS Study Group, J. Hern, E. Duff, A. Reynolds, D. Bartolanzo, K. Song, M. Milazzo, L. Francisco, S. Schech, A. Golway, T. Mebrahtu, E. Lee, K. Bohince, T. Hamm, K. Lombardi, M. Imbach, L. Eller, S. Peel, J. Malia, A. Kroidl, I. Kroidl, C. Geldmacher, C. Kafeero, A. Nambuya, J. Tegamanyi, H. Birungi, O. Mugagga, G. Nassali, P. Wangiri, M. Nantabo, P. Nambulondo, B. Atwijuka, A. Asiimwe, C.T. Nabanoba, M. Semwogerere, R. Mwesigwa, S. Jjuuko, R. Namagembe, E. Bagyendagye, A. Tindikahwa, I. Rwomushana, F. Ssentongo, H. Kibuuka, M. Millard, J. Kapkiai, S. Wangare, R. Mangesoi, P. Chepkwony, L. Bor, E. Maera, A. Kasembeli, J. Rotich, C. Kipkoech, W. Chepkemoi, A. Rono, Zeddy Kesi, Janet Ngeno, Edwin Langat, Keneddy Labosso, Ken Langat, Robert Kirui, L. Rotich, M. Mabwai, E. Chelangat, J. Agutu, C. Tonui, E. Changwony, M. Bii, E. Chumba, J. Korir, J. Sugut, D. Gitonga, R. Ngetich, S. Kiprotich, W. Rehema, C. Ogari, I. Ouma, O. Adimo, S. Ogai, C. Okwaro, E. Maranga, J. Ochola, K. Obambo, V. Sing’oei, L. Otieno, O. Nyapiedho, N. Sande, E. Odemba, F. Wanjiru, E. Chiweka, P. Kiliba, M. Mwaipopo, J. Muhumuza, N. Mkingule, O. Mwasulama, A. Sanagare, P. Kishimbo, G. David, F. Mbwayu, J. Mwamwaja, J. Likiliwike, J. Muhumuza, R. Mcharo, N. Mkingule, O. Mwasulama, B. Mtafya, C. Lueer, A. Kisinda, T. Mbena, H. Mfumbulwa, L. Mwandumbya, P. Edwin, W. Olomi, Y. Adamu, A. Akintunde, A. B. Tiamiyu, K. Afoke, S. Mohammed, N. E. Harrison, U. C. Agbaim, O. A. Adegbite, Z. Parker, G. A. Adelakun, F. O. Oni, R. O. Ndbuisi, J. Elemere, N. Azuakola, T. T. Williams, M. Ayogu, O. Enas, O. Enameguono, A. F. Odo, I. C. Ukaegbu, O. Ugwuezumba, S. O. Odeyemi, N. C. Okeke, L. Umeji, A. Rose, H. Daniel, H. Nwando, E. I. Nicholas, T. Iyanda, C. Okolo, V. Y. Mene, B. Dogonyaro, O. Olabulo, O. Akinseli, F. Onukun, G. Knopp

**Affiliations:** 1 HJF Medical Research International, Inc., Mbeya, Tanzania; 2 U.S. Military HIV Research Program, Walter Reed Army Institute of Research, Silver Spring, MD USA; 3 Henry M. Jackson Foundation for the Advancement of Military Medicine, Bethesda, MD USA; 4 National Institute for Medical Research-Mbeya Medical Research Center, Mbeya, Tanzania; 5 Muhimbili University of Health and Allied Science-Dar-Es-Salaam, Dar-Es-Salaam, Tanzania; 6 HJF Medical Research International, Kisumu, Kenya; 7 HJF Medical Research International, Kericho, Kenya; 8 Makerere University Walter Reed Project, Kampala, Uganda; 9 HJF Medical Research International, Abuja, Nigeria; 10 Memory and Aging Center, Department of Neurology, University of California, San Francisco, CA USA

**Keywords:** HIV and aging, Sub-saharan Africa, Elders on antiretroviral drugs, HIV treatment outcomes

## Abstract

**Introduction:**

With increased use of antiretroviral therapy (ART), HIV mortality rates are declining and people living with HIV (PLWH) are surviving longer. We characterized CD4 recovery and viral suppression among adults aged < 50 and ≥ 50 years living with HIV who initiated ART in the African Cohort Study (AFRICOS).

**Methods:**

Beginning in January 2013, PLWH at twelve clinics in Kenya, Uganda, Tanzania and Nigeria underwent medical history review, CD4 and viral load testing as part of the ongoing African Cohort Study (AFRICOS). ART-naïve PLWH who initiated ART within 30 days of enrollment and had at least one year of follow-up were included in these analyses. To compare ART response in participants < 50 years and ≥ 50 years old, changes in CD4 count and viral load suppression after ART initiation were examined at different time points using linear and binomial regression with generalized estimating equations. Variables for time since ART initiation and the interaction between age group and time on ART were included in the model to evaluate longitudinal changes in CD4 recovery and viral suppression by age.

**Results:**

Between January 2013 and September 2019, 2918 PLHV were enrolled in the cohort. Of these, 443 were ART naïve and initiated on ART within 30 days of enrollment, with 90% (n = 399) aged < 50 years old at ART initiation. At ART initiation, participants aged 50 and older had a higher median CD4 count compared to participants younger than 50 years of age although it did not reach statistical significance (306 cells/mm^3^, IQR:130–547 vs. 277cells/mm^3^, IQR: 132–437). In adjusted models examining CD4 recovery and viral suppression there were no significant differences by age group over time. By the end of follow-up viral suppression was high among both groups of adults (96% of adults ≥ 50 years old and 92% of adults < 50 years old).

**Conclusion:**

This study found no difference in long-term CD4 recovery or viral suppression by age at ART initiation. We found that particularly among younger adults participants had lower median CD4 counts at ART initiation, suggesting the importance of identifying and putting this population on treatment earlier in the disease course.

## Background

In Africa, HIV/AIDS has largely been considered a disease of young people due to high rates of HIV infection in younger populations[1, 2] and is the leading cause of death among young people in Africa and the second leading cause of death among young people worldwide [3]. However, with the advent of antiretroviral therapy (ART), persons living with HIV (PLWH) are living longer, the age distribution of the epidemic is shifting [4–8] and the life expectancy among PLWH and HIV-uninfected populations is relatively similar in recent years [9]. A study conducted in Kenya, Mozambique, Rwanda, and Tanzania found that the proportion of adults on ART aged 50 years and older increased from 10 to 16% between 2005 and 2010, primarily driven by aging of the existing patient population [10]. In 2016, approximately 2.4 million people aged 50 years and above were living with HIV in Sub-Saharan Africa [7]. This number is expected to triple by 2040 if treatment coverage remains widespread [3]. The major causes of morbidity and mortality among PLWH are no longer AIDS related, but are similar to people not living with HIV such as cancers, renal and liver diseases [11]. Many of the ongoing HIV interventions focus on younger age populations but it is important that HIV care and treatment programs consider the specific needs of the ageing population of PLWH as it continues to increase.

Ageing is a complex process affecting a wide variety of physiologic functions, including the development and maintenance of the peripheral immune system [12]. Most published data on immunologic responses to ART concern younger patients with very little published in older populations newly initiated on ART [13–15]. Evidence suggests that immune responses to HIV and ART may be different in older populations due to declines in thymic function leading to decreased thymic outputs and poor immunologic response to infections [16, 17]. Several studies of adults living with HIV and on ART suggest that differential immune responses are associated with thymus involution [14, 18, 19].

Data regarding the immunologic response to ART in older PLWH have been inconsistent. A study conducted in four African countries found that the 12-month average CD4 response for adults 50 years and older initiating ART was 20.6 cells/mm^3^ lower than for adults aged 25–39 years [20]. In contrast, several studies conducted in the United States did not find significant age-based differences in immune response after ART initiation. A clinic-based study in the United States that enrolled 906 ART naïve patients between February 1989 and January 2006 did not find significant differences in CD4 recovery after ART initiation by age when adjusting for CD4 count at the time of ART initiation [21, 22]. Similarly, an analysis of two large observational clinical cohorts in the US failed to observe significant differences in mean CD4 recovery after ART initiation between adults younger than 50 years and those 50 years and older [21, 23].

Conflicting findings have also been reported regarding viral load responses to ART initiation among adults. The majority of findings come from studies conducted in Europe and the US and report similar rates of viral suppression among adults aged < 50 years and those ≥ 50 years old [15, 24–26]. However, findings from other studies have revealed that older ART initiators presented with lower viral suppression rates compared to younger PLHIV ART initiators [11, 27] While several studies have examined immunologic and virologic response in HIV-infected older adults, few studies have focused on the long-term impact of age at ART initiation on CD4 recovery and viral suppression in African settings. We examined changes in CD4 recovery and viral suppression after first ART initiation, stratified by age, in a unique longitudinal cohort across four African countries.

## Methods

### Study design and participants

This analysis is nested within the African Cohort Study (AFRICOS), which was established in 2013 and has been described previously [28]. In brief, AFRICOS is an ongoing prospective cohort study enrolling PLWH at twelve clinical sites in Kenya, Tanzania, Uganda, and Nigeria. PLWH were eligible for AFRICOS if they were ≥ 18 years old, receiving HIV care at the enrolling PEPFAR clinic and consented to data and specimen collection. Individuals who were pregnant were not eligible for enrollment. The study was approved by the institutional review boards of the Walter Reed Army Institute of Research, the University of California San Francisco, the Makerere University School of Public Health, Kenya Medical Research Institute, the Tanzania National Institute of Medical Research, and the Nigerian Ministry of Defense. All participants provided written informed consent.

### Data collection and definitions

Demographic and clinical variables including sex, age, education level, comorbidities, nadir CD4 count, adherence to ART medication, type of ART regimen at ART initiation and clinical care site were collected upon enrollment. CD4 count and viral load tests were performed every 6 months. Viral suppression was defined as a viral load < 1000 copies/mL. To account for possible confounding effects, we included three noncommunicable diseases (NCDs) in our analyses: dysglycemia (fasting glucose > 99 or any glucose > 199 or on glucose medications), elevated blood pressure (systolic BP > 139 or diastolic BP > 89 or on hypertension medications), hypercholesterolemia (cholesterol > 199 or on cholesterol medications). Self-reported adherence to ART medication was assessed at each study visit. Participants were classified as adherent if they reported not missing any doses in the 30 days prior to the visit. We classified ART regimen into two categories based on the most common ART prescribed across the sites: on efavirenz/lamivudine/tenofovir, or on any other drug combination. We used a continuous CD4 measurement and viral suppression defined as viral load < 1000 copies/mL to determine treatment outcomes.

To ensure comparability of data across the sites, standard operating procedures for vital signs were followed and laboratory measures were performed at laboratories that were accredited by the College of American Pathologists or had successfully completed external quality assurance.

Data were entered and verified into an electronic data capture and management software (Clinplus platform;DZS Software Solutions, Bound Brock, NJ).

### Statistical analyses

ART-naïve PLWH who were enrolled as of September 1, 2019, initiated on ART within 30 days of enrollment and had at least one year of follow up time were included in these analyses. Participants were categorized into two groups by age at ART initiation: < 50 and ≥ 50 years based on the WHO/CDC definition for older age [29]. The association between age group and population-averaged CD4 count and virologic suppression was evaluated using linear and binomial regression with generalized estimating equations. An independent working correlation was included to account for repeated observations in the same individual. Variables for time since ART initiation and the interaction between age group and time were included in the model to evaluate the longitudinal changes in mean CD4 and virologic suppression by age stratum. Confounders were selected based on a 10% change in coefficients and variables classified as confounders for one outcome were locked into the model for subsequent outcomes. We restricted these analyses to the first 42 months of follow up to ensure adequate sample size at each time point.

In order to further assess response in CD4 we conducted a few additional analyses. First, we used logistic regression to estimate the odds of a 50 cell/mm^3^ increase in CD4 six months after ART initiation and a 100 cell/mm^3^ increase in CD4 12 months after ART initiation comparing the two age groups. Last, we used binomial regression with generalized estimating equations to estimate the odds of reaching a CD4 ≥ 500 cells/mm^3^ during the follow up period after ART initiation. All analyses were performed in Stata 15.0 (StataCorp, College Station, Texas).

## Results

### Cohort characteristics at enrollment

Between January 2013 and September 2019, 2918 PLHV were enrolled in the cohort. Of these, 443 were ART naïve and initiated on ART within 30 days of enrollment. The Kenyan sites constituted the plurality of participants (35.2%), followed by Tanzania (27.8%), Uganda (21.2%), and Nigeria (15.8%; Table 1). There was a significant variation by age, with 90% (n = 399) of participants aged < 50 years old at ART initiation. The median age of participants < 50 was 35.3 years (Interquartile range (IQR) 29.9—40.9 years) and 55.2 years (IQR 52.4–59.1 years) for those ≥ 50 years (*p* < 0.001). Females constituted 58.5% of all participants with 59.9% and 45.5% among < 50 and ≥ 50 years old respectively (*p* = 0.07). There was a statistically significant difference in the number of comorbidities recorded at enrollment among participants aged < 50 and ≥ 50 years, with fewer comorbidities among participants aged < 50 years (*p < *0.001). The majority (96%) of participants initiated ART on an efavirenz based regimen while 13 participants initiated ART on a nevirapine based regimen and three participants on a protease inhibitor. No participants initiated ART on an integrase inhibitor.

**Table 1:** Demographic and clinical characteristics of study participants at ART initiation

	All participants n = 443	< 50 years n = 399	≥ 50 yearsn = 44	*p *value
Study site				0.18
Kayunga, Uganda	94 (21.2%)	87 (21.8%)	7 (15.9%)	
South Rift Valley, Kenya	105 (23.7%)	90 (22.6%)	15 (34.1%)	
Kisumu West, Kenya	51 (11.5%)	47 (11.8%)	4 (9.1%)	
Mbeya, Tanzania	123 (27.8%)	108 (27.1%)	15 (34.1%)	
Abuja & Lagos Nigeria	70 (15.8%)	67 (16.8%)	3 (6.8%)	
Age at ART initiation (years), median (IQR)	36.4 (30.5, 43.2)	35.3 (29.9, 40.9)	55.25 (52.4, 59.1)	< 0.001
Gender				0.07
Male	184 (41.5%)	160 (40.1%)	24 (54.5%)	
Female	259 (58.5%)	239 (59.9%)	20 (45.5%)	
Education				< 0.01
None or some primary	118 (26.6%)	97 (24.3%)	21 (47.7%)	
Primary or some secondary	197 (44.5%)	186 (46.6%)	11 (25.0%)	
Secondary and above	128 (28.9%)	116 (29.1%)	12 (27.3%)	
Elevated blood pressure				< 0.001
No	401 (90.5%)	371 (93.0%)	30 (68.2%)	
Yes	42 (9.5%)	28 (7.0%)	14 (31.8%)	
Dysglycemia				0.63
No	367 (93.9%)	332 (94.1%)	35 (92.1%)	
Yes	24 (6.1%)	21 (5.9%)	3 (17.9%)	
Hypercholesterolemia				0.10
No	358 (89.7%)	326 (90.6%)	32 (82.1%)	
Yes	41 (10.3%)	31 (9.4%)	7 (17.9%)	
CD4 nadir, median (IQR)	259 (122.5, 389)	257 (119.5, 384.5)	326.5 (141, 545.5)	0.22
ART regimen at initiation				0.62
TDF/EFV/3TC	427 (96.4%)	384 (96.2%)	43 (97.7%)	
Other	16 (3.6%)	15 (3.8%)	1 (2.3%)	

*IQR* Interquartile Range, *TDF* tenofovir, *EFV* efavirenz, *3TC* lamivudine, *ART* antiretroviral therapy

### CD4 recovery

**Fig. 1:**
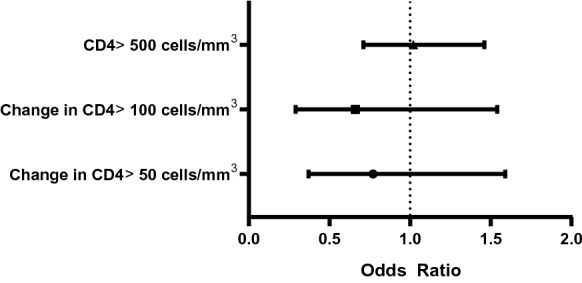
Odds ratios for achieving select CD4 cut-offs by time since ART initiation

At ART initiation, participants aged 50 and older had a higher median CD4 count compared to participants younger than 50 years of age (306 cells/mm^3^, IQR:130–547 vs. 277 cells/mm^3^, IQR: 132–437). After six months of follow up, median CD4 count increased in both age groups (≥ 50: 380, IQR: 183–702; < 50: 397, IQR: 226–583). In both unadjusted and adjusted models, there were no significant time and age interactions, suggesting no differences in mean CD4 recovery by age group over time (Table 2). There were no significant differences by age group in odds of a CD4 increase of 50 cells/mm^3^ at 6 months after ART initiation, 100 cells/mm^3^ at 12 months post-ART initiation, or achieving a CD4 count greater than 500 cells/mm^3^ during the course of follow up (Fig. 1).

**Table 2:** Unadjusted and Adjusted Change in CD4 for participants < 50 and ≥ 50 years by time since ART initiation

	Unadjusted change in CD4		Adjusted change in CD4^a^	
	Average CD4	95% CI	Average CD4	95% CI
Constant	306	283–330	282	218–345
Age ≥ 50	52	− 38–142	37	− 58–132
Time				
6 months	128	108–148	146	124–166
12 months	156	135–178	174	150–199
18 months	176	153–199	192	166–217
24 months	188	163–214	203	174–231
30 months	217	185–248	230	197–263
36 months	195	162–228	213	179–247
42 months	199	161–237	289	225–353
Age*visit interaction				
Age ≥ 50* 6 months	− 20	− 66–26	− 3	− 62–55
Age ≥ 50* 12 months	− 48	− 101–4	− 29	− 96–38
Age ≥ 50* 18 months	− 35	− 100–29	− 22	− 95–51
Age ≥ 50* 24 months	47	− 41–135	66	− 28–162
Age ≥ 50* 30 months	− 38	− 129–54	− 11	− 106–84
Age ≥ 50* 36 months	− 71	− 182–40	− 52	− 159–55
Age ≥ 50* 42 months	− 147	− 263–31	− 130	− 377–116

^**a**^Adjusted for study site, education, gender, dysglycemia and hypercholesterolemia

### Viral suppression

**Fig. 2:**
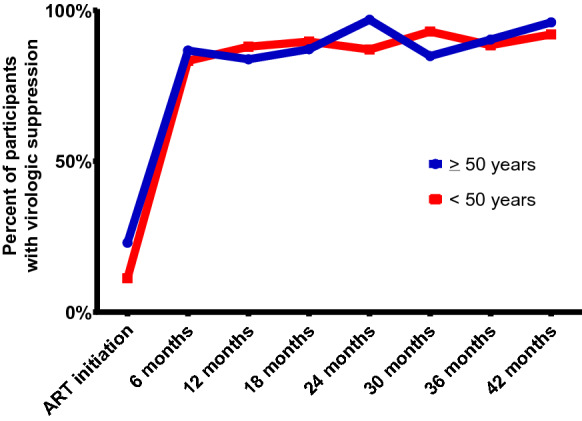
Proportion of participants virally suppressed over time, by age group

In both the unadjusted and adjusted model, there were no significant differences in viral suppression at any time point between the two age groups (Table 3). By the end of the follow-up period, at 42 months post ART initiation, viral suppression was high among both groups of adults, with 96% of adults ≥ 50 years old virally suppressed and 91% of adults < 50 years old virally suppressed (*p* = 0.43; Fig. 2).

**Table 3:** Unadjusted and Adjusted Odds Ratio for Viral suppression between participants < 50 and ≥ 50 years by time since ART initiation

	Unadjusted OR		Adjusted OR^a^	
	OR	95% CI	aOR	95% CI
Age				
< 50	Ref		–	
> 50	1.21	0.73–2.00	1.23	0.71–2.13
Time				
ART initiation	Ref		–	
6 months	4.29	3.56–5.17	4.38	3.53–5.43
12 months	4.29	3.56–5.16	4.48	3.65–5.50
18 months	4.26	3.54–5.13	4.26	3.43–5.30
24 months	4.16	3.45–5.02	4.30	3.49–5.29
30 months	4.33	3.59–5.23	4.50	3.64–5.56
36 months	4.34	3.60–5.22	4.39	3.56–5.40
42 months	4.50	3.73–5.43	4.74	3.82–5.89
Age*visit interaction				
Age ≥ 50* 6 months	0.82	0.49–1.39	0.78	0.42–1.47
Age ≥ 50* 12 months	0.88	0.53–1.45	0.84	0.49–1.44
Age ≥ 50* 18 months	0.83	0.50–1.37	0.86	0.49–1.53
Age ≥ 50* 24 months	0.92	0.55–1.52	0.90	0.52–1.57
Age ≥ 50* 30 months	0.85	0.51–1.41	0.80	0.45–1.44
Age ≥ 50* 36 months	0.86	0.50–1.47	0.84	0.46–1.51
Age ≥ 50* 42 months	0.81	0.46–1.42	0.86	0.50–1.49

^a^Adjusted for study site, education, gender, dysglycemia and hypercholesterolemia

## Discussion

We found that time since ART initiation rather than age was the primary driver of CD4 recovery and viral suppression among new ART initiators. Importantly, among adults of all ages, there was a large sustained increase in mean CD4 count and in the proportion of participants who were virally suppressed.

Though some other studies have documented that older PLWH initiating ART mount poorer CD4 cell responses than younger patients, our findings do not support these arguments but rather add to the growing body of evidence that immune recovery does not differ among older adult ART initiators [11, 18, 22, 26, 27]. A cohort study conducted in Italy among adult PLWH admitted between 1997 and 2003, found that older ART initiators can achieve the same immunologic recovery as younger ART initiators [10]. Another clinic-based cohort study conducted in the United States which followed 906 ART-naïve patients for a median duration of 46 months, found similar null findings after adjusting for CD4 count at the time of ART initiation [21, 22]. Other studies conducted in the US and Europe have failed to observe significant differences in CD4 response among adults aged < 50 years and those 50 years and older, after initiation of ART[21, 23]. This study extends similar findings in four countries in sub-Saharan Africa. In contrast, other studies have found differences in CD4 among older and younger individuals although these studies may differ from our findings due to the older population studied and differing ART initiation guidelines in practice at the time of the study [10, 30].

Our study found out that younger participants presented with lower mean CD4 counts at ART initiation compared to adults. This may be attributed to delayed testing due in part to a number of reasons including inadequate HIV and sexual education as well as lack of HIV services and stigma [3, 31]. Results from surveys conducted between 2011 and 2016 in sub-Saharan Africa showed that only 10% of adolescent boys and 15% of adolescent girls in sub-Saharan Africa had tested for HIV in the last 12 month [32]. However, although it was not statistically significant, we observed a trend toward faster immune response among younger participants compared to older participants in the acute phase of ART initiation. This is consistent with findings from the Multicenter AIDS Cohort Study (MACS) that revealed at acute phase of ART therapy, immune responses to ART were faster in younger groups compared to older groups, possibly due to bigger thymic size and active functionality, which become compromised with age [16, 17]. That younger PLWH have a faster initial immunologic response is likely outweighed in the long run by behavioral factors and poor ART adherence reported among young ART initiators [33].

We also characterized changes in viral suppression among ART naïve adults in this cohort. We found that both age groups responded well virologically to ART and sustained high levels of viral suppression for the duration of the follow-up period, over three years after initiating ART. Many studies conducted in Europe and the US found similar rates of viral suppression among adults aged < 50 years and those ≥ 50 years [15, 21, 24–26]. An analysis of a clinic-based cohort in France to evaluate the effect of age on ART efficacy and tolerance in 639 patients with HIV infection (99 aged < 50 years, and 540 aged ≥ 50 years) found similar virologic response among age groups after six months of follow-up [21]. In contrast, some studies have shown that older ART initiators reach viral suppression at a faster rate and maintain suppression for longer [22, 34–37]. A large cohort conducted in nine sites in Argentina, Brazil, Chile, Haiti, Honduras, Mexico and Peru with more than 26,000 participants found better viral suppression among adults 50 years and older as compared to those less than 50 years [38], which is likely due to differences in adherence rather than physiological reasons. Findings from an analysis of the US-based cohorts found that HIV-infected ART-naïve adults initiated on ART achieved virologic suppression faster than younger PLWH [13, 22, 27]. Increased viral suppression among adults 50 years and older may support the idea that older PLWH tend to avoid risk behaviors and have better ART adherence [24, 27, 39].

Strengths of this study include the long follow up time of 42 months for participants after initiation of ART. We also were able to capture information on other NCDs that may influence immune response, particularly among older participants. We acknowledge several limitations of this study. Many AFRICOS participants were already initiated on ART at enrollment limiting the sample size for these analyses, with a smaller proportion of adults ≥ 50 years of age compared to adults younger than 50 years (1:9), potentially allowing for outliers in the older age category to exhibit greater influence on mean CD4. The smaller sample size of participants ≥ 50 also limited our power to detect significant differences in mean CD4 between the two age groups. However, in the sensitivity analyses examining dichotomous CD4 the findings remained unchanged. Participants enrolled in AFRICOS receive an optimized care package and therefore may not be generalizable to the general care-seeking population. ART adherence was self-reported rather than measured by pill counts, which is prone to recall and social desirability bias. However, given the high percentage of participants virally suppressed after visit one we feel that participants were largely reliable in reporting good ART adherence. Large windows around study visits could potentially create imprecision around immune responses measured at different time points; however, as estimates of CD4 and viral load remained relatively constant at subsequent visits we feel these limitations had minimal impact. Lastly, participants are only seen every six months, so we were unable to assess changes in CD4 and viral load within a timeframe less than 6 months after ART initiation as other studies have [40, 41].

## Conclusion

This study found no difference in long-term CD4 recovery or viral suppression by age at ART initiation. Despite the fact that the difference was not statistically significant, we found that participants, particularly younger adults, had lower CD4 counts at ART initiation, suggesting the importance of identifying and putting this population on treatment earlier in the disease course.

## Data Availability

The Henry M. Jackson Foundation for the Advancement of Military Medicine (HJF) and the Water Reed Army Institute of Research (WRAIR) are committed to safeguarding the privacy of research participants. Distribution of data will require compliance with all applicable regulatory and ethical processes, including establishment and approval of an appropriate data-sharing agreement. To request a minimal data set, please contact the data coordinating and analysis center (DCAC) at PubRequest@hivresearch.org and indicate the RV329 study along with the name of the manuscript.

## Abbreviations

ART: Antiretroviral Therapy
HIV: Human Immunodeficiency Virus
WHO: World Health Organization
AIDS: Acquired Immune deficiency Syndrome
UNAIDS: United States Agency for International Development
AFRICOS: African cohort study
PEPFAR: President's Emergency Plan for AIDS Relief
RNA: Ribonucleic Acid
IRB: Institutional Review Board
PLWH: People Living With HIV
